# Neurotrauma—From Injury to Repair: Clinical Perspectives, Cellular Mechanisms and Promoting Regeneration of the Injured Brain and Spinal Cord

**DOI:** 10.3390/biomedicines12030643

**Published:** 2024-03-13

**Authors:** Andrew R. Stevens, Antonio Belli, Zubair Ahmed

**Affiliations:** 1 Institute of Inflammation and Ageing, University of Birmingham, Edgbaston, Birmingham B15 2TT, UK; a.belli@bham.ac.uk; 2 Centre for Trauma Sciences Research, University of Birmingham, Edgbaston, Birmingham B15 2TT, UK

**Keywords:** CNS, traumatic brain injury, spinal cord injury, neuroregeneration, neurotrauma, neuroprotection

## Abstract

Traumatic injury to the brain and spinal cord (neurotrauma) is a common event across populations and often causes profound and irreversible disability. Pathophysiological responses to trauma exacerbate the damage of an index injury, propagating the loss of function that the central nervous system (CNS) cannot repair after the initial event is resolved. The way in which function is lost after injury is the consequence of a complex array of mechanisms that continue in the chronic phase post-injury to prevent effective neural repair. This review summarises the events after traumatic brain injury (TBI) and spinal cord injury (SCI), comprising a description of current clinical management strategies, a summary of known cellular and molecular mechanisms of secondary damage and their role in the prevention of repair. A discussion of current and emerging approaches to promote neuroregeneration after CNS injury is presented. The barriers to promoting repair after neurotrauma are across pathways and cell types and occur on a molecular and system level. This presents a challenge to traditional molecular pharmacological approaches to targeting single molecular pathways. It is suggested that novel approaches targeting multiple mechanisms or using combinatorial therapies may yield the sought-after recovery for future patients.

## 1. Introduction

Trauma, a physical injury resulting from an external force, is ubiquitous across geographical and societal groups. Whilst many body tissues are capable of significant biological and functional repair, the human central nervous system (CNS) is not. In contrast to the peripheral nervous system (PNS), CNS neurons do not replicate to replace cells lost after injury, and surviving neurons are not capable of regenerating their axons [1]. Due to the unique and profound functions of the CNS, the ramifications of neurotrauma without recovery are of enormous significance to individuals, their families and wider society. Amongst several possible and valid definitions of neurotrauma, the present review will consider neurotrauma as: “traumatic injury to the brain or spinal cord”.

Neurotrauma is an enormously heterogeneous disease state, with a variety of possible clinical and biological phenomena that occur after the initial insult. Even the simple anatomical dichotomy between injury to the brain and spinal cord can be undermined by the increasing incidence of comorbid injury to both, termed “tandem” neurotrauma. Beyond this, there are infinite permutations of injury biomechanics and comorbidities, as well as a broad spectrum of clinical severities and differing relative burdens of discrete deleterious post-injury biological mechanisms. As such, an appreciation of the varying clinical contexts and management strategies is important in understanding the complexities involved in developing therapeutics for promoting functional recovery. As such, the following review will first describe the overall importance, classification, pathophysiology and clinical management of traumatic brain injury (TBI) and spinal cord injury (SCI). The molecular and cellular basis of neurotrauma in general will then be described, given the considerable commonalities between TBI and SCI, and this will form the basis of a discussion on contemporary approaches to promoting neural repair and regeneration after injury.

## 2. Traumatic Brain Injury

### 2.1. Importance

TBI is a significant global health challenge, with no disease-modifying treatment shown to improve outcomes. A principle cause of morbidity and mortality in young adults, the incidence of TBI in Europe is estimated at 1012 cases per 100,000 people per year and 939 per 100,000 globally [1,2]. TBI disproportionately affects low-to-middle-income countries and is a significant financial burden to economies worldwide; the total annual cost globally is estimated to be in the region of £47 billion [3,4]. The risk of suffering TBI is present across society, and injuries are often sustained through road traffic collisions, falls or assault. A wide range of life-changing sequelae may result from injury, including: motor and sensory deficits, cognitive dysfunction, impaired consciousness, depression, behavioural changes, and increased mortality, including an increased risk of suicide [5,6,7,8,9]. These consequences are as yet untreatable beyond supportive therapy in the acute setting, and rehabilitative therapy thereafter [10].

### 2.2. Classification

TBI is typically stratified by either symptomatic severity or anatomical measures. The clinical presentation of a patient can be used to stratify severity based on the Glasgow Coma Scale (GCS) [11], where a GCS score of 13–15 is mild, 9–12 is moderate and ≤8 is severe [12]. The duration of loss of consciousness or post-traumatic amnesia can also be used to classify injury severity [13]. Anatomical measures can be used, for example, to classify TBI by location of haemorrhage, presence of diffuse axonal injury or the presence/absence of multiple variables, as in the computerised tomography (CT)-derived Marshall or Rotterdam grading systems [14,15]. Emerging alternative stratification tools, for example, using immunohistochemical markers, are in their early research phases and are not yet widely accepted. The differences in outcomes, management and pathophysiology vary enormously across this spectrum of injury sub-types. Though termed “mild” TBI (also termed concussion), the long-term consequences can have a severe impact on quality of life and ability to function [5,6,7,8,9]. Mild TBI accounts for up to 90% of TBI, and persistent symptoms occur in up to one-third of people. Severe TBI is invariably a life-changing injury, with mortality rates as high as 40%, and often results in long-term significant disability [5].

### 2.3. Pathophysiology

The damage resulting from trauma to the brain is typically considered in two parts: ‘primary’ and ‘secondary’. ‘Primary brain injury’ is sustained by the immediate event of trauma itself, whilst ‘secondary brain injury’ occurs after injury due to a variety of adverse sequelae resulting in further cell death and damage [16]. Though primary injuries are modifiable (through personal and public health measures to reduce the incidence and severity of injuries), acute medical care interventions typically focus on the mitigation of secondary injury mechanisms as modifiable targets to improve patient outcomes. Secondary injury to neural tissue can occur through a broad variety of mechanisms, spanning cellular, systemic and anatomical processes. On a cellular level, mitochondrial dysfunction after injury can result in metabolic failure and oxidative stress, which have a role in the propagation of injury and trigger apoptotic cell death after TBI, with associated effects on long-term function [17,18]. “Metabolic crisis” is a phenomenon in TBI that results in severe metabolic dysfunction despite adequate provision of metabolic substrates [19]. Neuroinflammatory processes, whilst essential for wound healing and restoration of the blood–brain barrier (BBB), result in the harmful propagation of injury into the penumbra (areas of the brain with lesser injury, surrounding an injury focus). This principally involves microglial activation: a predominance of M1 (proinflammatory) over M2 (pro-repair) phenotypes within populations of these resident tissue macrophages of the CNS [20].

The initial injury and ensuing necrosis result in the dysregulated release of neurotransmitters (such as glutamate) [21]. Their activation of local synapses leads to uncontrolled regional depolarisation, known as excitotoxicity. Locally, this can compound metabolic dysfunction and result in regional dysregulated cellular activation (cortical spreading depolarisation) or global seizures [22,23]. The development of cerebral oedema and the expansion of surgical mass lesions, result in increasing intracranial pressure (ICP). Due to the fixed volume of the intracranial space, expansion of intracranial contents results in increasing ICP, as first described in the Monro–Kellie hypothesis [24]. This can result in a variety of harmful sequelae and is the prevailing cause of mortality in the early phase after TBI. Anatomically, this can lead to compression (and obfuscation in extremis) of arteries, cranial nerves and ultimately brain parenchyma. Where this includes compromise of the delicate structures of the brainstem, including regions of respiratory control, mortality rates are high. Increasing ICP also results in global compromise of cerebral blood flow, with unfavourable effects on brain oxygenation and the provision of essential metabolites (predominantly glucose), which further propagate cellular dysfunction after injury.

Whilst the pathophysiology of TBI has been the subject of many years of scientific research, the processes that propagate neural damage after injury are not fully elucidated. More recent observations have been attributed to supporting emerging hypotheses of further injury mechanisms. For example, damage to the BBB through injury may result in dysregulated entry of systemic molecules into the CNS, such as pro-inflammatory cytokines. Similarly, the dysregulated release of adenosine triphosphate (ATP) from damaged neurons has been hypothesised to contribute to increased neuroinflammation and cellular apoptosis [25].

### 2.4. Clinical Management

Whilst there is a growing understanding of the pathophysiological mechanisms of TBI, the opportunities to measure and correct these processes are limited. Contemporary therapeutic paradigms in severe TBI are predominantly based on the monitoring of ICP, informing clinical decision-making to offer intervention for correction and normalisation of these indices as supportive measures [26]. ICP monitoring is typically performed using a temporarily implantable fibre optic probe, which is placed within the brain parenchyma to a depth of 1.5–2 cm. This probe connects externally to a transducer and a user interface, allowing real-time pressure readings to be presented to the clinician. The procedure to implant an ICP probe requires a hole to be drilled through the skull, and an opening in the dura and cortex is made via a sharp puncture. The entry point is typically at Kocher’s point (an anatomical point 11 cm posterior to the nasion in the mid-pupillary line). A plastic self-tapping, hollow bore “bolt” is screwed into the skull hole to house and secure the ICP wire. Similarly, placement of an intraventricular catheter (external ventricular drain (EVD)) can be used to monitor intracranial pressure via the transduction of pressure within the ventricle.

Acute management of TBI is predominantly targeted at control of ICP alongside general supportive intensive care and management of other traumatic injuries. Whilst this paradigm has been the subject of some historical debate, ICP remains at the centre of clinical guidelines [26]. Therapeutic interventions to reduce ICP use are as follows: (1) patient head positioning, (2) therapeutic hypocapnia, (3) sedation and paralysis, (4) osmotic therapy, (5) diversion of cerebrospinal fluid (CSF), (6) barbiturate-induced coma and (7) decompressive craniectomy [26,27,28,29,30,31]

**Figure 1:**
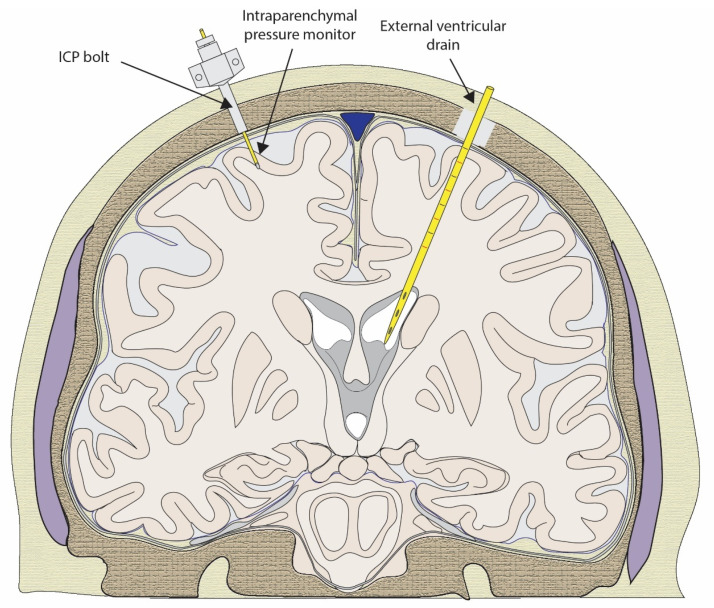
Schematic diagram of a coronal section of the head with an intraparenchymal pressure monitor (with bolt) and external ventricular drain in situ. Both devices may be used to measure pressure from their respective compartments via a transducer.

Since the establishment of ICP monitoring as a standard of care in TBI, additional monitoring capabilities have been integrated with an ICP probe into cranial access to increase the scope of the “bolt” paradigm (Figure 1). This has seen the greatest success with the introduction of partial pressure of brain tissue oxygen (PbtO_2_) monitoring and, to a lesser extent, with microdialysis probes [32,33,34]. The extent to which the inclusion of these technologies provides information that is of value for clinical decision-making is yet to be conclusively determined, and as such, they are variably implemented. Despite this, early investigations of PbtO_2_ monitoring have shown a trend towards lower mortality with its use, and it has been included in TBI guidelines for ensuring adequate cerebral oxygenation during hyperventilation therapy [26,35]. Beyond these invasive monitoring techniques, there are a few established methods to identify and monitor the pathological processes that occur after injury [36,37,38]. As such, there are limited opportunities to direct targeted therapies at specific secondary mechanisms.

## 3. Spinal Cord Injury

### 3.1. Importance

Traumatic SCI is damage to the spinal cord sustained by mechanical trauma, resulting in a deficit in neurological function [39]. Injuries affect people across society and the globe, with a bimodal age distribution. Injury may occur from falls, road traffic collisions, or sporting accidents, and less commonly from assaults and penetrating or blast injuries [40]. SCI is of increasing prevalence, with 2500 new cases occurring each year in the UK alone [41], resulting in additional lifetime costs of around £2.8 billion each year [41,42]. The lifelong disabilities caused by SCI are typically profound: loss of motor and sensory function, loss of bladder, bowel and sexual function, as well as neuropathic pain and, in some cases, tetraplegia and loss of respiratory function [39]. Whilst some recovery can be anticipated in incomplete injuries through rehabilitation, the loss of function sustained in SCI is typically permanent, as the spinal cord, like the rest of the CNS, has no innate capacity for repair [43].

### 3.2. Classification

SCI is most commonly classified using the American Spinal Injury Association (ASIA) impairment scale [44], which classifies injury based on neurological impairment, measured by a thorough and standardised International Standards for Neurological Classification of Spinal Cord Injury (ISNCSCI) clinical examination [44]. This identifies whether an injury is “complete” (i.e., no preservation of any neurological function below the level of injury) or “incomplete” (i.e., partial preservation of motor, sensory or sacral function below the level of injury), as well as identifying the neurological level of injury (the lowest (most caudal) spinal cord segment with intact neurological function). Both the neurological level and severity of the injury indicate the prognosis for functional outcome [45]. The severity of injury has been correlated with the likelihood of recovering the ability to walk independently, with complete injury (ASIA A) associated with the lowest probability of independent ambulation (Table 1) [45,46].

**Figure 2:**
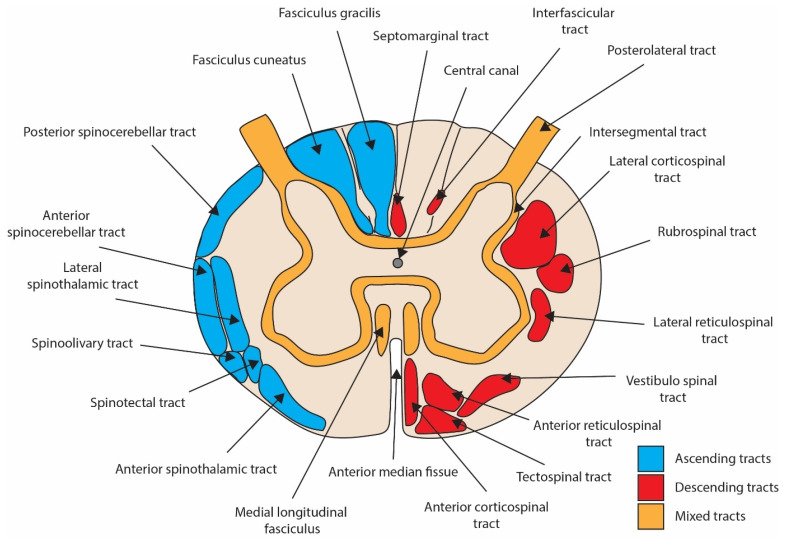
Schematic diagram of an axial cross-section of the spinal cord with labelled ascending, descending and mixed tracts (structures exist bilaterally).

**Figure 3:**
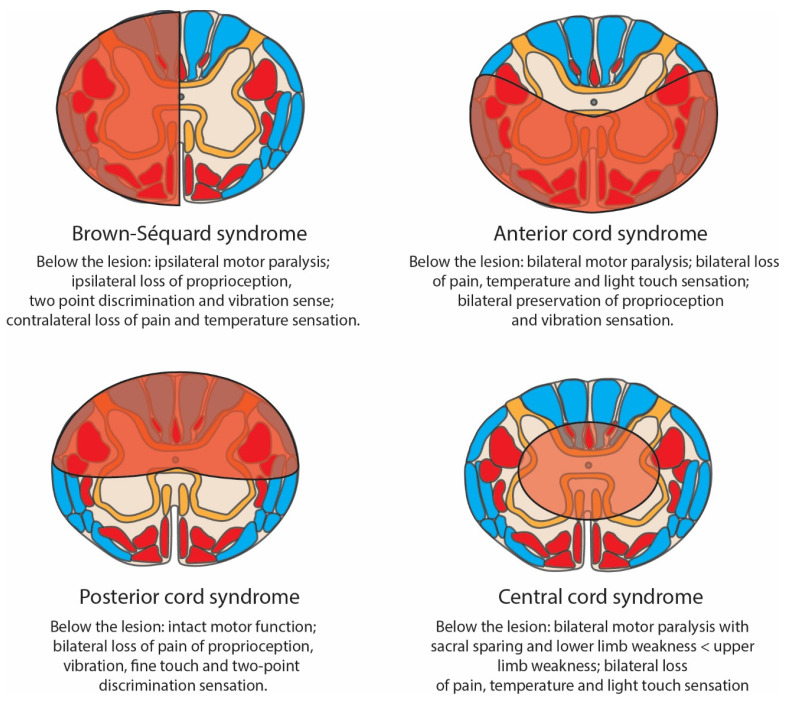
Schematic diagram of four classically described incomplete spinal cord injury syndromes with brief descriptions of their typically associated clinical features. Damaged regions are denoted in translucent red. Motor tracts = opaque red; sensory tracts = blue. Brown-Séquard syndrome (hemisection of the cord) can occur following trauma, particularly penetrating injuries, or from the expansion of tumours. Anterior cord syndrome can occur during trauma or ischaemia. Posterior cord syndrome typically follows posterior spinal artery occlusion. Central cord syndrome is a cervical SCI that can occur after a hyperextension injury with pre-existing cervical stenosis.

The spinal cord is a complex structure, composed of grey matter (unmyelinated) and white matter (myelinated). Three columns can be recognised as structures of the cord, which run bilaterally in a rostro-caudal plane: the dorsal, ventral and lateral columns (Figure 2). Within these columns are more focal tracts, categorised as ascending (afferent/sensory), descending (efferent/motor) and mixed (Figure 2). SCI can be classified by spinal cord regions damaged/affected by the injury and may be complete (with all tracts affected) or incomplete (with some tracts preserved) [46,47,48]. The regions affected and corresponding clinical presentations of incomplete SCI syndromes are shown in Figure 3. Similarly, the variety of pre-clinical models for the study of traumatic SCI result in differing neurological deficits dependent on the anatomical location of the injury.

### 3.3. Pathophysiology

The pathophysiological phenomena of SCI, as an acute traumatic insult to the tissues of the CNS resulting in swelling within an enclosed and fixed bony space, hold many similarities to those described above for TBI. Similarly, the injury mechanisms can be considered “primary” (occurring directly from trauma) and “secondary” (as subsequent events and consequences following the primary injury) [39,45,49]. Primary injury can result from direct spinal cord trauma (from penetrating objects or primary blast trauma) or, more commonly in civilian settings, from mechanical force and pressure from fracture and/or dislocation of the surrounding spinal column. Bony displacement, fragments, or the resultant haemorrhage can all mediate primary injury [39,45,49]. Though primary injury typically occurs at the time of the trauma, the primary neuronal injury can be delayed from the index traumatic event: trauma that compromises the mechanical stability of the spinal column can result in delayed mechanical injury to the spinal cord only after weight-bearing [39,49].

The initial trauma to the spinal column and spinal cord commences a complex cascade of secondary injury mechanisms, as seen in TBI [39,45]. In the acute post-injury phase, vascular or bony injury can compromise arterial supply to the spinal cord, resulting in prolonged ischaemia and ongoing neuronal injury, whilst resultant haemorrhage can cause direct pressure effects with compressive effects on local tissue [39,45]. Dysregulated necrotic release of neurotransmitters such as glutamate from neurons and astrocytes can result in excitotoxicity, intracellular calcium influx and ultimately cell death via apoptosis or necrosis, accompanied by sodium influx resulting in oedema [39,45,50,51]. The release of reactive oxygen species (ROS) and free radicals from necrotic or dysfunctional cells can result in oxidative stress and lipid peroxidation [49,52]. Metabolic failure further contributes to this ionic and oxidative disturbance [52,53]. Damage to the blood-spinal cord barrier (BSCB) disturbs its protective function and allows unregulated migration of inflammatory cells and cytokines into the area to perpetuate the local inflammatory response, contributing to local spinal cord oedema, which can in turn result in further damage [39,54,55].

In the sub-acute phase, there is ongoing apoptotic activation within and surrounding the umbra of the injury site [56]. Growth cone collapse and aborted axonal regeneration, along with demyelination and continuation of the inflammatory response that initiates glial scar formation, ensue [43,57,58]. In the chronic phase, cavitation and maturation of the glial scar, along with degeneration and regression of the remaining axons, occur [45].

### 3.4. Clinical Management

In further similarity to TBI, current therapeutic paradigms for SCI focus primarily on the mitigation and prevention of secondary damage, particularly via mechanical and hypoxic damage [39,49]. Initial assessment and management follow Advanced Trauma Life Support guidelines and involve resuscitation for maintenance of spinal perfusion pressure [39,59,60]. Immobilisation of the spinal column for resuscitation is recommended where possible to avoid additional damage through the mechanical effects of instability [61]. More definitive management of bony instability is typically achieved via fixation, accompanied (where indicated) by bony decompression of the spinal cord [39,62,63,64,65]. Along with the supportive management of blood pressure dysregulation (through neurogenic shock, orthostatic hypotension and autonomic dysreflexia), targeted blood pressure regulation and augmentation to optimise spinal cord perfusion is also a common feature of contemporary management [66,67]. Management beyond these targeted therapies is supportive, managing the complications of injury and promoting functional recovery through physical therapies and rehabilitation [39].

## 4. Molecular and Cellular Responses to Neurotrauma

The general principles of promoting survival and repair of the CNS neuron after traumatic injury can be considered across both TBI and SCI. All CNS neurons do not regenerate once injured, and typically enter apoptosis or a senescent state. Lost or dysfunctional neurons cannot be replaced through proliferation since neurons are post-mitotic. The resulting immediate cell loss and damage (primary injury) from the direct effects of the trauma are not modifiable once sustained. As such, current interventions in neurotrauma care aim to mitigate secondary injuries. In TBI, optimisation of ICP decreases early mortality and mitigates pressure-induced brain injury. In SCI, spinal decompression and fixation may mitigate the propagation of secondary injury through pressure effects or prevent subsequent mechanical injury through bony instability. Despite much research into neuroprotection and neuroregeneration, however, no therapeutic intervention is presently available that improves functional outcomes through promoting survival or repair of neurons after injury. Long-term rehabilitation may improve functionality, facilitated through neural plasticity and the use of physical aids. However, the capacity for functional recovery by these means is extremely limited at present, particularly in severe injuries, owing largely to the innate failure of the CNS to repair or regenerate neurons.

CNS responses to injury can be categorised into three phases, which are not entirely distinct but represent the principal processes occurring over general time periods after injury [68]:

1. Acute phase (I) (0–3 days post-injury);
2. Subacute phase (II) (3–14 days post-injury);
3. Chronic/consolidation phase (III) (14 days onwards post-injury).

### 4.1. The Acute Phase

#### 4.1.1. Haemorrhage

Along with direct traumatic injury to neural tissue and necrosis of directly damaged cells, the index traumatic event results in damage to local blood vessels, leading to haemorrhage into the injury site [39]. This, if of large volume, can result in not only direct compressive effects but also the delivery of cytokines, blood-derived immune cells (lymphocytes, neutrophils and macrophages), clotting factors and growth factors into the injured neural tissue, usually excluded by the blood–CNS barrier. Activation of the coagulation cascade and platelet degranulation results in the release of transforming growth factor beta (TGFβ) and platelet derived-growth factor (PDGF) [69,70].

#### 4.1.2. Inflammatory Cascade

**Figure 4:**
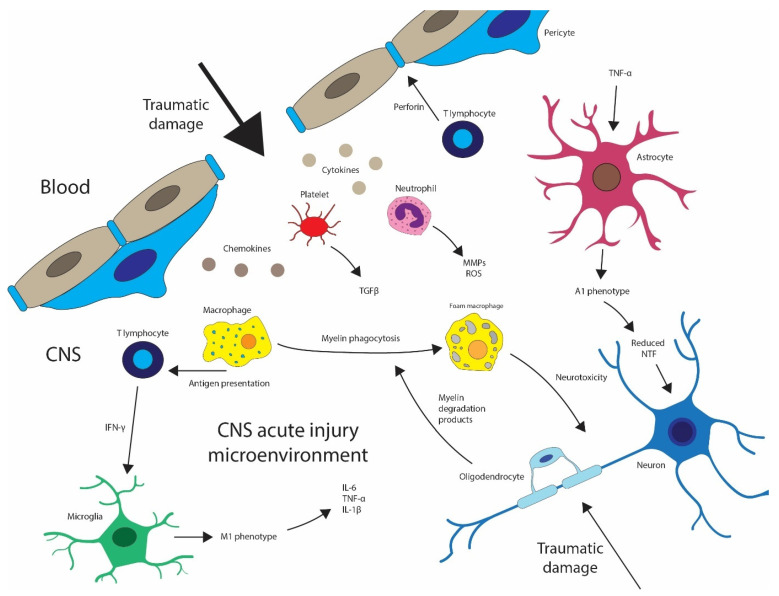
Schematic diagram of the acute phase of injury, following compromise of the blood–CNS barrier and entry of blood-derived cells and signalling proteins, detailing pro-inflammatory signalling leading to microglial/astrocytic polarisation.

The presence of TGFβ and proinflammatory mediators activates the inflammatory cascade, directly and indirectly via chemokine release (activating migrated blood-derived immune cells (macrophages, neutrophils and lymphocytes) and resident glia (microglia and astrocytes)) (Figure 4). This activation potentiates the inflammatory response, releasing further TGFβ, as well as interleukins (ILs) (IL-1α/β, IL-2, IL-6 and IL-8 [71]) and epidermal growth factor (EGF) [70]. Local chemokines also result in tissue remodelling via upregulation of matrix metalloproteinases (MMPs) and plasminogen activator 1 (PA-1). Whilst the initial neuroinflammatory response is triggered during this acute phase, the immune response persists throughout these three phases [68]. To an extent, leucocyte activation is favourable in traumatic injury for the restoration of blood–CNS barrier integrity and wound sterilisation and debridement. Neutrophils sterilise the wound of foreign pathogens by phagocytosis, with further debridement and the release of inflammatory mediators, MMPs and ROS. Monocytes deposit extracellular matrix (ECM) and initiate angiogenesis via the release of vascular endothelial growth factor (VEGF). Macrophages and resident microglia have a multi-faceted role, with favourable effects (mitigating local excess neurotransmitters from necrotic release, tissue remodelling and growth factor release) and unfavourable effects (myelin phagocytosis, demyelination and astrogliosis).

Populations of microglia, the resident macrophages of the CNS, demonstrate a biphasic response after trauma, with peaks within acute/sub-acute as well as chronic consolidation phases [68,69,72]. However, of greater importance than microglial presence in a favourable or unfavourable environment post-injury is their phenotype. Microglia may become polarised to an M1 phenotype (pro-inflammatory) or an M2 phenotype (anti-inflammatory, pro-repair) [20,69,73,74]. Microglia in the M1 state are understood to release pro-inflammatory cytokines/chemokines, including TNFα, IL-6 and IL-1β and increase surface expression of cluster differentiation (CD)16, CD32, CD40 and CD86 (Figure 4). Conversely, M2 phenotype microglia increase expression of CD163 and CD206, producing anti-inflammatory mediators (IL-10), growth factors (insulin-like growth factor-1 (IGF-1), fibroblast growth factor (FGF)) and neurotrophic factors (e.g., nerve growth factor (NGF) and brain-derived neurotrophic factor (BDNF)) [74,75]. In addition to well-characterised inhibitors/mediators of inflammation, there are emerging microglial-neuronal crosstalk mechanisms such as direct synaptic interfaces, extracellular vesicles and communication via gap junctions. Together, these suggest a complex and pivotal role for microglia in neurotrauma pathophysiology [76].

Macrophages (monocyte-derived) exhibit similar M1/M2 phenotypes as microglia, with corresponding pro- and anti-inflammatory roles within the CNS after injury. M1 phenotypic switching in SCI has been related to the presence of extracellular myelin [77], present in abundance in the context of axonal disruption within white matter tracts. Macrophages, alongside microglia, clear myelin and other cellular debris from necrosis and axonal shearing by phagocytosis, with greater macrophage residence in the lesion core and greater microglia accumulation within the penumbra. Foam macrophages, derived from macrophage phagocytosis of myelin, can result in paradoxical damage once formed [69]. Influx of peripherally circulating T lymphocytes similarly can promote microglial (M1) activation through the release of interferon-gamma (IFNγ) [78] and perpetuate increased permeability of the blood–CNS barrier through the release of perforin [79]. Conversely, T cell responses to myelin basic protein (MBP) have been associated with chaperoned microglial phenotype shifting to M2 [80].

#### 4.1.3. Compromise of the Blood–CNS Barrier

Loss of integrity of the blood–CNS barrier from the immediate trauma, sustained by the molecular activity described above by T-lymphocytes, amongst other mechanisms, permits continued compromise of the exclusion of the CNS from the blood-derived immune cells and circulating inflammatory mediators, which in turn sustains the neuroinflammatory response and contributes to developing oedema. This process is illustrated in Figure 4.

#### 4.1.4. Excitotoxicity

**Figure 5:**
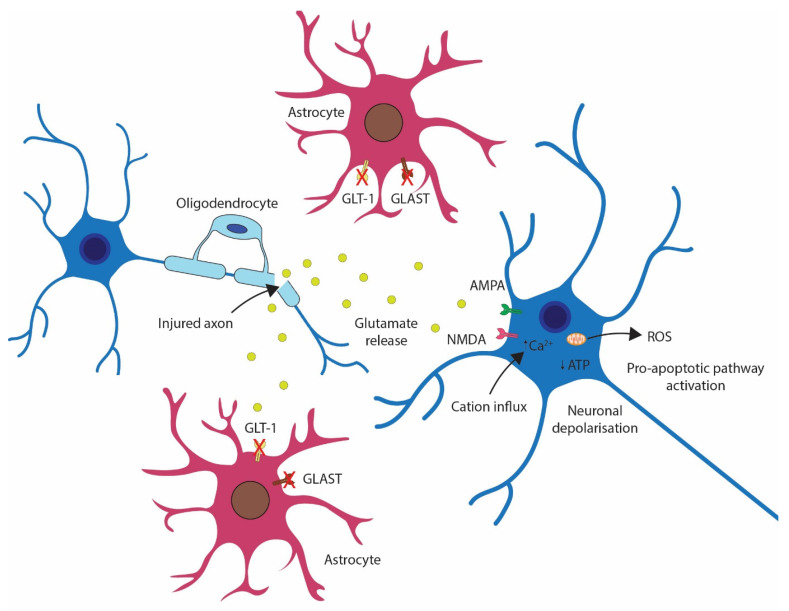
Schematic diagram of the excitotoxicity resulting from uncontrolled glutamate release from severed pre-synaptic axons, perpetuated by downregulation of astrocytic capacity for scavenging free glutamate.

Excitotoxicity results from the increased and uncontrolled release of excitatory neurotransmitters after trauma, principally glutamate [16,68]. Glutamate release from damaged axons in the spinal cord and pre-synaptic terminals in the brain results in accumulation within the injury microenvironment [21], compounded later by impaired reuptake due to decreased astrocytic expression of glutamate transporters glutamate aspartate transporter (GLAST) and glutamate transporter (GLT)-1 [81]. Glutamate activates α-amino-3-hydroxy-5-methyl-4-isoxazolepropionic acid receptor (AMPA) and N-methyl-D-aspartic acid (NMDA) receptors, which permit influx of cations (K^+^, Na^+^, and Ca^2+^) and depolarisation, with excessive activation resulting in intracellular Ca^2+^ accumulation, compromising mitochondrial function [16], contributing to ROS production and activating apoptotic pathways. This process is illustrated in Figure 5.

#### 4.1.5. Oedema

Oedema in neurotrauma is a significant mechanism of secondary injury propagation, which occurs through three mechanisms. Cytotoxic oedema is a result of the failure of ATP-dependent Na^+^-K^+^ pumps (particularly in astrocytes), resulting in the accumulation of Na^+^ (and consequently, water via aquaporin water channels and the G protein-coupled receptor, GPRC5B [82,83,84,85]) within the cell. Ionic oedema follows, with the diffusion of Na^+^ ions across the intact blood–CNS barrier into the extracellular space to replenish those sequestered intracellularly by cytotoxic oedema. Vasogenic oedema occurs through the influx of water and solutes across a compromised blood–CNS barrier (particularly large proteins such as albumin) into the interstitium of the CNS.

### 4.2. Sub-Acute Phase

Axonal sprouting: initial early axonal sprouting of damaged neurons after injury can be observed in the early sub-acute phase after injury. This is later aborted, as the initial modest release of neurotrophic factors after injury is not sustained. The generation of a non-permissive injury microenvironment via other mechanisms inhibits any remaining drive for growth from residual neurotrophic factors. This is in contrast to peripheral nervous system injury, where Schwann cells produce a consistent and graded concentration of neurotrophic factors to support axonal regeneration [86,87].

#### 4.2.1. Astrocyte Activation

Astrocytes, the multifunctional support cell of the CNS, become activated after traumatic injury, resulting in their proliferation within the lesional area, transformation to “reactive” astrocytes (astrogliosis) and upregulation of the expression of glial fibrillary acidic protein (GFAP) [88]. Reactive astrocytes have two characterised phenotypes: A1 proinflammatory/neurotoxic astrocytes and A2 anti-inflammatory/pro-survival astrocytes (akin to the phenotypic polarisation of reactive microglia) [16,69,89,90]. A1 astrocytes are formed via the activation of the nuclear factor kappa-light-chain-enhancer of activated B cells pathway (induced by microglial secretion of IL-1α and TNFα) and secrete an uncharacterised neurotoxin that triggers neuronal and oligodendrocyte cell death [90,91] (Figure 4). Expression of component C3 is used to identify A1 astrocytes [69]. A2 astrocytes were initially identified as being polarised by ischaemic injury and are specifically induced via TNFα/IL-1β/IL-6-mediated activation of the signal transducer and activator of transcription 3 (STAT3) pathway [89,92,93]. Scar formation via A2 astrocytes can create a more permissive environment for regeneration through an astroglial scar [94], with A2 astrocytes playing a role in increasing the availability of neurotrophins [91]. A2 astrocytes may be identified by their specific expression of S100A10, pentraxin-3 (PTX3), S1Pr3 and Tweak [69,95].

#### 4.2.2. Initiation of the Glial Scar

Immediately after injury, a lesion core is formed through haemorrhage as a collection of non-neuronal cells, blood products, CSF and serous fluid that accumulate through the damaged blood–CNS barrier. Astrocytes migrate to the periphery of this core lesion site and begin to form a network of tightly connected peripheral processes to surround and corral the lesion core to effect a physical barrier between the lesion core and the penumbral neural tissue [69]. Fibroblasts (cells that form connective tissue) from dura/blood and pericytes (endothelial cells of capillary networks and blood–CNS barrier interfaces) also migrate to this zone and proliferate [69,96]. Upon forming a network, astrocytes, pericytes and fibroblasts begin to form an ECM with the secretion of laminin, collagen (type IV), fibronectin and chondroitin sulphate proteoglycan (CSPG), which form the molecular meshwork of the glial scar [69,88]. Whilst the presence of the glial scar, particularly the presence of CSPG, forms both a physical barrier and a non-permissive chemical microenvironment, the presence of an ECM appears necessary for axonal regeneration, with matrix proteins such as laminin acting as an intercellular skeleton, as total scar suppression impairs stimulated axonal regeneration [94].

#### 4.2.3. Demyelination

Acute damage to myelin can occur either by direct damage to the myelin sheath itself (alongside axonal injury) or due to damage to the supporting oligodendrocyte from which the myelin is derived [16,68,97]. The damaged myelin also contributes to this non-permissive microenvironment via the release of inhibitory proteins such as myelin-associated glycoprotein (MAG), neurite outgrowth inhibitor-A (Nogo-A) and oligodendrocyte-derived myelin glycoprotein (OMgp). Akin to the mechanisms by which neurons are lost after trauma, oligodendrocytes and their associated myelin can undergo continued damage during the subacute and chronic phases post-injury: excitotoxicity, oxidative stress, inflammatory cytokines and necrotic proteolytic enzymes. The role of lymphocytes in immune amplification is favourable for the response to pathogens; however, they form endogenous myelin-reactive lymphocytes, initiating immune-driven demyelination that potentiates CNS damage [98]. Furthermore, oligodendrocytes appear dependent on neuronal survival, and axonal degeneration and neuronal apoptosis result in further loss of oligodendrocytes [97].

#### 4.2.4. Mitochondrial Dysfunction

Mitochondrial dysfunction after neurotrauma is a mechanism of secondary injury across cell types, with exquisite effects on the neuron due to its high metabolic demands. Rises in intracellular Ca^2+^, typically due to excitotoxicity or oxidative stress, act as an initiator of mitochondrial crises. Mitochondrial Na^+^/Ca^2+^ exchange channel activity permits Ca^2+^ entry into the mitochondria, with rising intra-mitochondrial calcium leading to opening of the mitochondrial permeability transition pore (mPTP), mitochondrial oedema and swelling, loss of mitochondrial membrane potential and severe disruption of ATP synthesis [99,100,101]. This membrane damage results in the release of mitochondrial proteins such as cytochrome c, Ca^2+^ and reactive oxygen species (ROS) into the cytosol, which in turn can trigger apoptosis [101]. Additionally, an upregulation in the activity of nitric oxide synthase (NOS) and an increase in nitric oxide (NO) production can independently impair electron transport chain (ETC) function [101].

#### 4.2.5. Oxidative Stress

**Figure 6:**
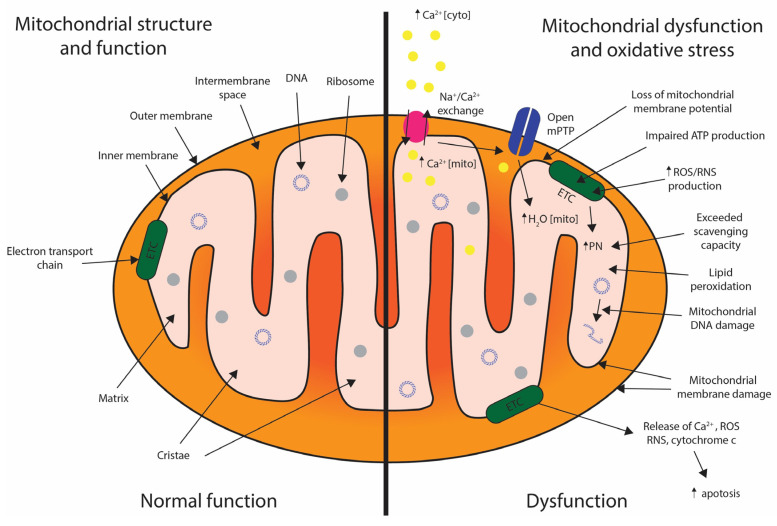
Schematic diagram of mitochondrial structure and normal function (**left**) and dysfunction after trauma (**right**). Dysfunction here is triggered by rising cytosolic Ca^2+^ (Ca^2+^ cyto), resulting in increased mitochondrial Ca^2+^ (Ca^2+^ mito). This opens mPTP channels and intramitochondrial oedema, loss of mitochondrial membrane potential, impaired ATP production, an increase in ROS/RNS production and the release of mitochondrial pro-apoptotic proteins into the cytosol.

Dysfunctional mitochondrial activity results in the release of ROS, or free radicals. In states such as neurotrauma, where ROS and RNS production is confluent, production far outstrips any antioxidant/scavenger capacity [99,101]. Mitochondrial production of nitric oxide (NO) and electron leakage from the electron transport chain (ETC) to produce superoxide radicals (O^−2^) result in the formation of peroxynitrite (PN). PN and other potent oxidising agents propagate mitochondrial damage via lipid peroxidation, leading to mitochondrial DNA damage [99]. This mediates further disruption of the mitochondrial structure, allowing the release of ROS, which results in the destruction of cellular structures, proteins and lipids, triggering apoptotic pathways, the release of pro-inflammatory mediators and perpetuating secondary injury [68]. Mitochondrial structure, function and dysfunction after neurotrauma are illustrated in Figure 6.

### 4.3. Consolidation Phase

#### 4.3.1. Apoptosis

Triggered by a variety of stimuli in the post-injury tissue environment, the loss of CNS cells can persist in the chronic phase after injury due to apoptosis. Apoptosis, as a controlled process of programmed cell death, contrasts with the disordered events of necrosis, which are typical of immediate traumatic cell death in the acute phase [102]. Apoptosis may occur either as an intracellular (intrinsic) stress response, mediated by Bcl-2/Bax, or due to extracellular factors (extrinsic) in response to a variety of local cell signalling molecules [56,102,103,104]. Intrinsic pathways to apoptosis may be triggered via a variety of the mechanisms discussed, including mitochondrial dysfunction, oxidative stress, lipid peroxidation and excitotoxicity [91]. Intrinsic and extrinsic pathways activate a series of intracellular signalling pathways mediated by the cysteinyl aspartic proteinases (caspase) family. Caspases are grouped into “initiator” caspases (Caspase-8, -9 and -10) and “executioner” caspases (Caspase-3, -6 and -7), whilst caspase-2 shows activity across both functions (Caspase-2) [104,105].

#### 4.3.2. Consolidation of Glial Scar

**Figure 7:**
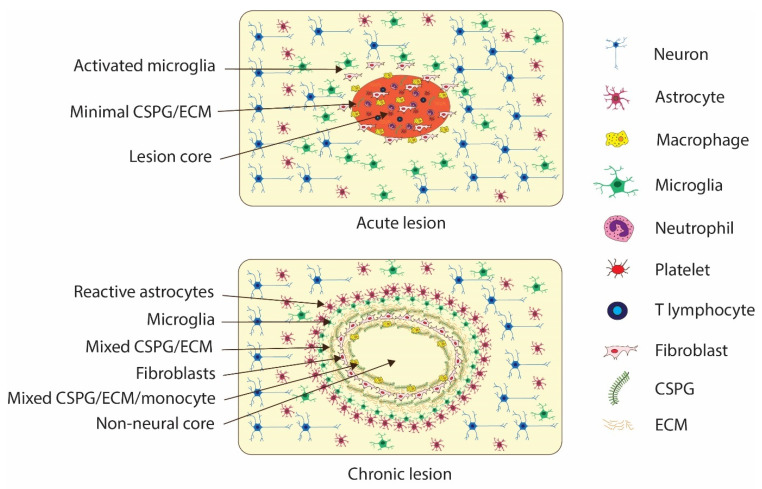
Schematic diagram of the acute phase (**top**) and chronic/consolidation phase (**bottom**) of glial scar formation and cavitation, with description of cellular properties of layers from the lesion core to the penumbral neural tissue.

Consolidation of the glial scar during the chronic phase after injury creates a continual inhibitory environment for neurons attempting to regenerate severed axons. In SCI, cavitation, that is, fluid-filled cysts, expands over a period of months as inflammatory cells remove non-viable tissue, and an expanding zone of apoptosis, degeneration and demyelination occurs [106]. The acute and consolidation phases of glial scar formation are illustrated in Figure 7.

#### 4.3.3. Aborted Axonal Regeneration

In the non-permissive milieu of the tissue microenvironment post-injury, perpetuated by the consolidation of the glial scar, there is a combination of an abundance of inhibitory factors and a scarcity of neurotrophic factors. Low concentrations of neurotrophins are insufficient to promote or maintain axonal regeneration in the context of non-permissive factors [86,87]. Myelin-derived inhibitory factors continue to contribute to the collapse of the axonal growth cone. MAG, Nogo-A and OMgp bind to the Nogo receptor (NgR) complex (composed of toxicity and JNK inducer (TAJ), p75 neurotrophin receptor (p75^NTR^) and either leucine-rich repeat and immunoglobin-like domain-containing protein 1 (LINGO-1) or amphoterin-induced gene and open reading frame-3 (AMIGO-3) [107,108] and activate an intracellular pathway mediated by Rho-A and Rho-associated protein kinase (ROCK) [16]. Via their respective receptors, non-myelin-derived signalling molecules such as ephrins [109] and semaphorins (semaphorin 3A [110]) also converge on this pathway to inhibit cofilin activity and promote growth cone collapse [16,111].

Neurotrophins (such as nerve growth factor (NGF), brain-derived neurotrophic factor (BDNF), neurotrophin 3/4 (NT-3/4), transforming growth factors (TGFs), fibroblast growth factor 2 (FGF2), epidermal growth factor (EGF), vascular endothelial growth factor (VEGF), transforming growth factor β1 (TGF-β1), glial cell line-derived neurotrophic factor (GDNF) and insulin-like growth factors (IGFs)) act predominantly at the tropomyosin receptor kinase (Trk) receptor family (A/B/C) via various intracellular signalling pathways [112,113,114,115]. The transmembrane protein p75^NTR^ can interact directly with low affinity for NTFs, potentiate NTF affinity at Trk receptors or bind pro-neurotrophins at the sortilin receptor [112]. Co-activity of p75^NTR^ in the presence of NTFs mitigates its activity in the NgR complex, reducing the effect of inhibitory signals.

## 5. Models and Organisms Used for the Study of Neurotrauma and Regeneration

Contemporary understanding of the cascade of biological events that occur in the aftermath of trauma to the CNS is a composite of insights generated from over a century of research, derived from a broad range of in vitro and in vivo models across species, as well as observations from clinical studies. A full description and analysis of the respective advantages of these approaches is beyond the scope of the present review, but they are described briefly below as a short summary, principally to highlight key limitations in the deployment of these models in advancing understanding of neurotrauma and regeneration in humans. For further reading on these topics, the reader is directed elsewhere [116,117,118,119,120,121,122,123].

### 5.1. In Vitro Models

#### Experimental Models

In vitro models are advantageous in some respects to the study of trauma and regeneration, offering high reproducibility and throughput at a relatively low cost. Specific cell types may be studied in isolation or in combination through 2D/3D co-culture/scaffold/organoid models. The use of organotypic tissue slice cultures also offers an in vitro model that mimics the composition and cell:cell interactions of their expected state in vivo [122,123]. Application of traumatic injury modelling to these cultural paradigms allows a precise study of the specific effects of mechanical forces (or their ensuing sequelae). Common methodologies to model traumatic injury in vitro are as follows:

- Compression: direct impact via weight drop or pendular acceleration [122,123].
- Stretch: distortion of a culture membrane or other substrate, transmitted to the adhering cells or tissue. A multitude of variables are possible (uniaxial stretch, biaxial stretch, shear, etc.) [122,123].
- Transection: scratching or other sharp distortion of cells/tissue, usually perpendicular to the orientation of axons [122,123].
- Static pressure: a high-pressure chamber to replicate the conditions of raised ICP [124].
- Chemical: application of adverse biochemical conditions to simulate the post-injury microenvironment, for example, oxidative stress, oxygen-glucose deprivation, serum withdrawal, excitotoxicity, etc. [122,123].
- Whilst these methods offer some advantages, observations of cell isolates or co-culture constructs in vitro may be markedly different from those observed in vivo. As well as the general differences in behaviour of cells in vitro as compared with in vivo, this is also attributable to the roles of a broad range of cell types and contributions from a diverse array of system-level adverse conditions (e.g., raised local tissue pressure, regional ischaemia, cortical spreading depolarisation and migrating inflammatory/progenitor cells). As such, whilst in vitro investigation has a significant role in the understanding of neuroregeneration, there is an inherent risk of artefactual observations, the possibility of which must always be considered.

### 5.2. In Vivo Models

#### 5.2.1. Species

The response to traumatic injury and the intrinsic regenerative capacity of the CNS varies greatly across the animal kingdom. Within vertebrates, some injurious responses differ: for example, whilst glial scarring occurs post-SCI across mice, rats and humans, only the spinal cord of the mouse does not undergo cavitation after injury and demonstrates increased post-injury angiogenesis [125]. As such, in vivo injury modelling in mammalian species has informed much of the contemporary understanding of how the human CNS responds to traumatic injury. Lower-order vertebrates, such as some species of bird, fish or amphibian, display significant contrast from mammalian species in response to CNS injury, by demonstrating capacity for significant or complete CNS repair after trauma.

Interest has been shown in the intrinsic capacity of the zebrafish (*Danio rerio*) for CNS repair [120]. In stark contrast to the events following mammalian injury, described above, in the zebrafish, ependymo-radial glial cells (ERGCs) proliferate and migrate to the lesion site and provide “bridging” support to guide regenerating axons from the ends (“stumps”) of axons cleaved during injury. Astrocyte activation and the ensuing astrogliosis are not observed. Some mechanisms, such as activation of apopotic pathways and oxidative stress, are also common to zebrafish [126]. Owing to the vastly differing cellular populations, genetic differences and differing neuronal responses, such work carries inherent limitations. However, neuroregeneration research using zebrafish has identified novel mechanisms and elucidated detail on the role and function of some potential therapeutic targets, such as: neuropeptide Y [127], MMP-9 [128], caveolin 1 [129] and the role of lipid droplets and the TAR DNA-binding protein of 43 kDa (TDP-43) in regulating microglial activation [130].

The amphibian species Xenopus (*Xenopus laevis*) has been investigated due to its regenerative capacity during the larval stage, which is lost after metamorphosis [131]. During larval stages, injury results in significant proliferation of neural stem progenitor cells (NSPCs) and the absence of glial scarring, and complete regeneration is observed at 20 days post-injury. In the mature Xenopus, however, deposition of ECM proteins (fibronectin and collagen) and an absence of proliferation more closely represent mammalian injury responses and, similarly, result in a consolidated chronic scar without neuronal regeneration. Research has further identified a key role of JAK/STAT pathway activation within Sox2/3^+^ ependymal cells and a key role for Sox2/3^+^ NSPCs in mediating the juvenile Xenopus regenerative response [132,133].

Key phylogenetic differences between such species and humans may have thus far limited the translational potential of some targets ascertained through such studies, though these models offer a contrasting means to study the mechanisms of non-regeneration in mammals and may generate important insights or genetic targets through ongoing work. Furthermore, the relatively high throughput possible with such species and the possibilities of transgenics may lead to further future impact on the understanding of non-regeneration through the use of these models, in combination with studies in mammalian models.

#### 5.2.2. Experimental SCI Models

**Figure 8:**
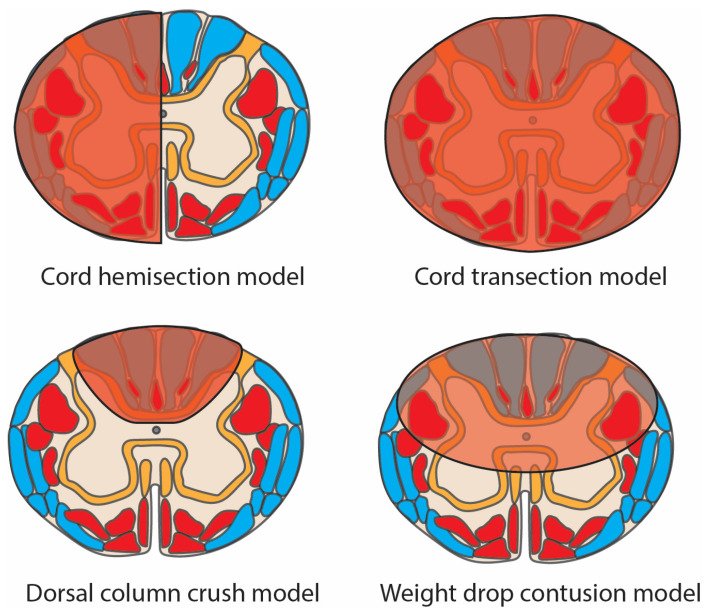
Schematic diagram of four pre-clinical spinal cord injury models used in the literature to replicate the conditions of traumatic injury. Damaged regions are denoted in translucent red. Motor tracts = opaque red; sensory tracts = blue.

The biomechanics of SCI in humans varies greatly owing to a complex array of variables, often in association with the type of force exerted by the varying deformation of the surrounding spinal column during (or persisting/occurring after) the injurious event. Various experimental injury methodologies have sought to replicate this in vivo [45,117]. These are summarised below, with examples illustrated in Figure 8.

- Compression (affecting modifiable anatomical regions, as shown in Figure 8): this is typically performed using either aneurysm clips [134], calibrated forceps [135] or an inflatable balloon catheter [136].
- Contusion: controlled impact on the spine or spinal cord by mechanical impact by a weight or driven by pressure [45,117].
- Transection: complete disconnection, usually via sharp dissection, of rostro-caudal segments, either partial (often hemisection [137]) or complete cord transection [138].
- Distraction: application of tension force along the axis of the spinal cord [45,117].
- Dislocation: displacement of one vertebra against an adjacent vertebra, resulting in shear force along the axis of the spinal cord [45,117].
- Whilst a variety of models is valuable for the study of differing responses to SCI subtypes, this can impede the relevance of findings made through the use of any one discrete model. For example, whilst cord hemisection (Figure 8) closely mirrors the Brown-Séquard syndrome described in humans (Figure 3), this phenomenon after traumatic injury is rare and usually only observed occasionally after stab injury.

#### 5.2.3. Experimental TBI Models

**Figure 9:**
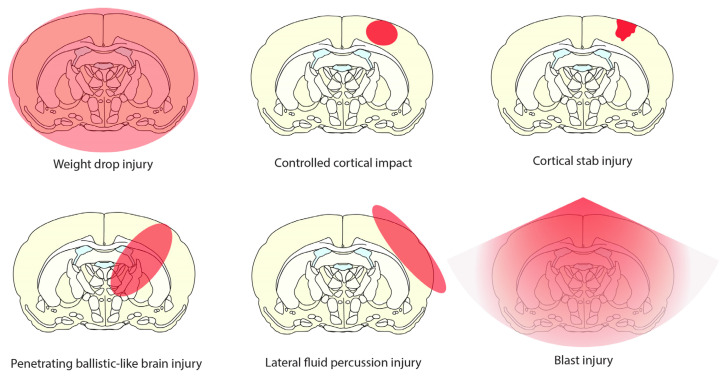
Schematic diagram of six pre-clinical traumatic brain injury models used in the literature to replicate the conditions of traumatic injury.

A variety of methodologies for administering traumatic injury to the brain have been employed in the study of the mechanisms and therapeutics of TBI and regeneration [116,139,140,141]. These can be considered diffuse or focal injury models. Diffuse models, weight drop (WD) or blast injury result in damage across the brain, with severity dependent on the magnitude of the exerted force, mainly resulting in diffuse axonal injury (DAI) within white matter tracts such as the corpus callosum (Figure 9) [142]. Blast injury modelling, via sound wave propagation and differential impedance at tissue/fluid interfaces, results in a specific injury pattern distinct from WD [116]. These are both closed injury models, where the skull is left intact. Focal injury methods target a more specific area of the brain. Controlled cortical impact (CCI) is induced by a metal- or silicone-tipped rod driven by a piston into the cortical surface. Cortical stab injury, either through the skull or following craniotomy, is performed with a controlled injury delivered by typically a scalpel [141]. Lateral fluid percussion injury is delivered by a pendulum device that “percusses” a volume of sterile fluid onto the cortical surface, which somewhat diffuses the injury across a wider area than CCI [116,139,140,141]. Penetrating ballistic-like brain injury utilises the inflation of a balloon catheter inflated after cannulation of the brain to a subcortical depth. This is designed to mimic the cavitation effect of a ballistic injury [143].

Whilst experimental reproducibility is advantageous, TBI in humans (regardless of military or civilian populations) is often heterogeneous, and an individual case will often encompass features of both diffuse and focal injury. Direct-to-cortex methods (such as LFPI or CCI) require a craniotomy to administer, which has the disadvantage that the injury site has undergone a bony decompression prior to the brain injury. This will inherently alter the local response to injury and oedema and effectively represent a pre-emptive therapy analogous to a small decompressive craniectomy.

## 6. Approaches to Promoting Neuroprotection and Neuroregeneration

The pathophysiological mechanisms described above contribute significantly to the failure of the CNS to survive and regenerate after injury. The current understanding of neuronal regeneration describes key features of this phenomenon:

1. Insufficient and unsustained provision of neurotrophic factors after injury;
2. Neuronal/glial apoptosis;
3. Formation and consolidation of a glial scar;
4. Release of local inhibitory factors from migrating and resident immune and glial cells;
5. Collapse of growth cones of regenerating axons;
6. Rarity of establishing functional reconnections with targets distal to the injury.

Effective therapies to improve functional neurological recovery therefore need to address two broad pathophysiological mechanisms: (1) the propagation of secondary injury via the multitude of mechanisms that contribute to further cell loss and the creation of a microenvironment that is strongly inhibitory of regeneration; and (2) the promotion of axonal regeneration and the establishment of functional reconnections. A multitude of approaches have been utilised in therapeutic attempts to mitigate damage (neuroprotection) and/or promote repair (neuroregeneration) after traumatic injury to the CNS through means to intervene with the pathophysiological mechanisms described above.

The progress of potential pharmacological agents has met difficulties in recent decades. Methylprednisolone remains a controversial therapeutic option. The original publication of the National Acute Spinal Cord Injury Study (NASCIS2) in 1990 resulted in widespread implementation of methylprednisolone therapy in SCI on the basis of unclear and inconsistent results, and its inclusion in clinical guidelines has been conflicting in the period since [144,145]. More recent attempts to validate any beneficial effects of SCI have not provided conclusive evidence [145,146,147]. Administration of methylprednisolone in TBI has been demonstrated in the CRASH trial to increase the risk of two-week mortality [148]. There is current interest in the early administration of gabapentinoids to promote functional recovery after SCI [149,150]. Despite some encouraging results from early human studies, this is yet to be confirmed in prospective clinical trials [151,152,153]. Riluzole, a glutmatergic modulator approved for use in amyotrophic lateral sclerosis (ALS), was deemed promising as a neuroprotective therapy, though the trial was terminated due to enrolment challenges, and the results are awaited [154,155]. The investigation of riluzole in TBI is ongoing [156].

Research is ongoing to optimise control of ICP and intra-spinal pressure (ISP) in the acute phase after injury as an indirect means of neuroprotective therapy via mitigation of secondary injury as a consequence of pressure effects on neural structures. Following the establishment of ICP control as a therapeutic target in severe TBI [26], intraspinal pressure and perfusion have been an area of growing interest in SCI. Direct pressure monitoring with targeted therapy, perfusion pressure optimisation and dural decompression (expansion duroplasty) has been proposed to mirror the pressure-directed surgical care in TBI [157,158,159]. A study is presently ongoing to assess the potential therapeutic benefits of expansion duroplasty in the acute phase after SCI, with the rationale of creating additional intraspinal volume than bony decompression alone, in order to permit post-injury oedema and limit local pressure effects. Based on a similar rationale, lumbar drainage of CSF has been proposed as a strategy to achieve more favourable intraspinal pressure, with some early success in pre-clinical studies [160]. Aligned with optimisation of pressure control, ensuring appropriate perfusion of the brain and spinal cord after traumatic injury is a further area of ongoing research for neuroprotection [158,161].

## 7. Discussion

Whilst molecular targets and novel approaches hold some promise for promoting repair and recovery after neurotrauma, pharmacological methods typically target single receptors and affect discrete pathways within the complex and multifaceted pathophysiology of the CNS after injury. Novel therapies targeting a variety of the pathophysiological processes in neurotrauma remain a significant area of research, as comprehensively described elsewhere [16,43,69,162]. These are summarised in Table 2. Whilst a number of biological targets have proven promising in pre-clinical studies, translational success has often proven challenging [49,83,106,107,163,164,165,166,167,168,169,170,171,172,173,174,175,176,177]. Interventional studies continue to investigate novel targets and approaches but have thus far failed to prove efficacious in improving functional outcomes [16,69,178,179]. This may be attributable to the intrinsic limitation of targeting single pathways in a disease process mediated by a multitude of factors. Efforts to improve future outcomes from neurotrauma therefore focus upon opportunities to intervene with the breadth of harmful cellular mechanisms, including monitoring their progression to provide targeted treatment. Combinatorial therapies may present a possible route to greater efficacy. An example of this is the success demonstrated through the combination of stem cell approaches with hydrogel scaffolds [180,181]. Exploration of targeting multiple pathways or using a multitude of approaches described in Table 2 may address this challenge. The potential for drug–drug interactions present a challenge to the potential strategy of combination therapies, which target multiple pathways and mechanisms to overcome the multitude of barriers to repair described above, or through the use of drug therapies combined with approaches such as biomaterials or CNS stimulation through devices.

The heterogeneity of TBI and SCI is itself a challenge. Compounding the enormous range of injury severities and classifications, the clinical outcomes from similar-severity injuries (based on unmeasurable variables or genomic idiosyncrasies) introduce further variability into studies that strive to improve functional outcomes. Consequently, clinical efficacy studies require large numbers of recruited patients to demonstrate benefit. The financial expense and high rate of failure of such studies have undoubtedly impacted the translational study of approaches that have proven promising in discovery science.

A broad range of biomarkers of CNS injury and injury severity have been identified, which can be readily measured in biofluids [182,183,184,185], though these are not recognised as markers of neuroregeneration. Some may be involved in neuroregeneration, for example, CSF concentrations of NGF [186,187]; however, there is insufficient evidence at present to posit these as a regeneration marker (as opposed to a marker of injury severity alone). In contrast, there are a number of specific protein markers for the identification of neuroregeneration (e.g., GAP43 [188], collapsin response mediator proteins [189] and genomic markers [190]), though these are only used in immunohistochemical analysis or next-generation sequencing of neural tissue, rendering them unsuitable for clinical applications. As such, there remains a reliance on clinical evaluations of functional outcomes, which can only be reliably measured years after injury and are subject to many other (known and unknown) variables. Advances in the availability of biomarkers of regeneration may provide much-needed early validation of the therapeutic efficacy of the interventions in clinical trials to allow real-time recognition of successfully induced neuroregeneration.

A focus on a dichotomy of favourable and unfavourable outcomes presents a challenge: therapeutic strategies must overcome a great threshold to increase the proportion of patients achieving a “favourable” outcome across a population. However, marginal gains in additional function for those severely injured can greatly improve quality of life. Short time frames of follow up compound this challenge, as recovery may continue well beyond the three- or six-month end points of typical neurotrauma clinical studies. The development of efficacy biomarkers (proxy indicators of recovery that are valid in early phases) may allow greater confidence in therapeutics to be gained in small pilot studies and is suggested as an area for further research. Other advances, for example, in patient stratification based on emerging techniques, may improve possibilities for novel study designs to improve the sensitivity of clinical studies to detect patient benefits or to personalise targeted interventions based on the individual burden of the secondary injury mechanisms discussed above [23,34,191,192,193,194,195].

**Table 2:** Summary of therapeutic approaches for neuroprotection and neuroregeneration. This is an illustrative list encompassing some common therapeutics under current and recent investigation. For further details on current clinical trials, see elsewhere for a comprehensive discussion of TBI [179] and SCI [196]. NGF = nerve growth factor; BDNF = brain-derived neurotrophic factor; IGF-1 = insulin-like growth factor 1; CS = chondroitin sulphates; PEDF = pigment epithelium-derived factor; Rho-A = Ras homolog family member A; mTOR = mammalian target of rapamycin; chk2 = checkpoint kinase 2; NgR = Nogo-66 receptor; AQP-4 = aquaporin 4; mPTP = mitochondrial permeability transition pore; ADSCs = adipose-derived stem cells; DPSCs = dental pulp stem cells; ESC = embryonic stem cells; IL-6 = interleukin-6; iPSC = induced pluripotent stem cells; NSC = neural stem cells; NPC = neural progenitor cells; MSC = mesenchymal stem cells; nNOS = neuronal nitric oxide synthase; OPC = oligodendrocyte progenitor cells; PLGA = poly (lactic-co-glycolic acid); siRNA = small interfering ribonucleic acid; HDAC = histone deacetylase; Uqcr11 = ubiquinol-cytochrome c reductase, complex III subunit XI.

Biological	Neurotrophic factors Pathway inhibitors Cell death inhibitors Receptor inhibitors Channel inhibitors Inflammation Mitochondria Oxidative stress Glial scar Gene therapies Autophagy Endocrine Other	NGF [172], BDNF [173], PEDF [135] and IGF-1 delivery via nanofibrous dural substitutes [197] Caspases [174], Rho-A [175], mTOR [176], chk2 [177], Rab [198] and transglutaminases [199] Caspases [174], Bcl-2 [200], imipramine [201], cyclosporin A [202] and statins [203] NgR [107], glutamate [163] and endothelin [204] AQP-4 [83], Ca^2+^ channel inhibitors [164] and mPTP [165] Immunomodulation [166], gangliosides [49,167], HDAC inhibitors [205] and bexarotene [206] Mitochondria-endoplasmic reticulum contact sites [207] Antioxidants [168], ROS scavenger materials [170,171,208,209] and Uqcr11 overexpression [210] Chondroitinase ABC [169,170], decorin [106,171] and 4-methylumbelliferone [211] Neuronal differentiation [43,212] HSPs [213] Progesterone [214], erianin [215] Hydrogen sulphide [216], tetramethylpyrazine [217], zinc [218], probucol [219], phenserine tartrate [220] and hyperbaric oxygen [221]
Cell therapies	Stem cells Neural cells Immune cells Advanced cell therapies	ESCs [222], iPSCs [43,223], NSCs/NPCs [224,225], MSCs [180,181], OPCs [226], DPSCs [216] and ADSCs [227] Olfactory ensheathing cells [228] and Schwann cells [229] Microglia [230] Directly reprogrammed NPCs (drNPCs) [231,232,233]
Gene therapies	Nucleic acid-based therapies Delivery methods Other	siRNA to AQP-4 [234], nNOS [235], iNOS [236], IL-6 [237], claudin-5 [238], RhoA [239,240], PLK-4 [241], PTEN [242,243], Sema3A [244], CTGF [245], combinatorial [246] and in combination with MSCs [242] Nanoparticle-coated siRNA [247,248,249], polymer nanocarriers [239], exosome delivery [243,245] extracellular vesicles [250], intrathecal delivery [240], photomechanical wave [251] and intranasal delivery [242] Chemogenetic stimulation [252]
Biomaterials	Porous polymers Natural polymers Nanoscaffolds Nerve guidance Other	Hydrogels [180,181,253,254], PLGA [255] and PLA [256] Collagen [181,257], CS [258], silk [259,260], decellularised ECM [227], modified gelatine [261] R-GSIK [262], electrospun nanofiber nets [263] and gene scaffolds [264] Gold nanoparticle nerve guidance conduits [265] and collagen conduits [266] Graphene oxide [267], IGF-1 delivery via nanofibrous dural substitutes [197] and ROS scavenger materials [170,171]
Physical	Stimulation Neuromodulation Supportive	Electrical [268,269], magnetic [270,271], ultrasound [272,273], light (photobiomodulation) [274,275] and combinatorial [276] Spinal stimulators [277] in combination with task training [278] Exoskeletons [279,280] and neuroprosthesis [281]

## 8. Conclusions

Developing new, effective therapies to avert the profound and permanent functional impacts of neurotrauma is an area of urgent need. The complexities of the post-injury micro- and macro-environments are described here, which span multiple intracellular pathways and cell types and encompass phenomena on intra- and inter-cellular levels (such as metabolic) and at a system level (such as the effects of impaired perfusion and increased pressure), suggesting that multifaceted approaches to improving outcomes will be required. Exploration of methods to target multiple mechanisms of injury propagation and consolidation may yield novel, effective interventions, which may offer a step-change in opportunities to rescue and restore function of the CNS after trauma.

## Figures and Tables

**Table 1:** Likelihood of independent ambulation after one year (positive predictive value (PPV)) with 95% confidence interval (CI) based on ASIA impairment scale classification (based on van Middendorp et al., 2011 [46]).

ASIA Grade	PPV	95% CI
A	8.3%	5.2–12.6
B	39.4%	27.6–52.2
C	61.8%	50.0–72.8
D	97.3%	92.2–99.4

## Data Availability

No new data were created in the preparation of this review.
